# Prenatal and neonatal factors involved in the development of childhood allergic diseases in Guangzhou primary and middle school students

**DOI:** 10.1186/s12887-019-1865-0

**Published:** 2019-12-07

**Authors:** Bolan Yu, Lijuan Dai, Juanjuan Chen, Wen Sun, Jingsi Chen, Lili Du, Nali Deng, Dunjin Chen

**Affiliations:** 1 Key Laboratory for Major Obstetric Diseases of Guangdong Province, The Third Affiliated Hospital of Guangzhou Medical University, No.63 Duobao Rd, Guangzhou, 510150 China; 2 Guangdong Engineering and Technology Research Center of Maternal-Fetal Medicine, The Third Affiliated Hospital of Guangzhou Medical University, No.63 Duobao Rd, Guangzhou, 510150 China; 3 Health Promotion Centre for Primary and Secondary Schools of Guangzhou Municipality, Guangzhou, China

**Keywords:** Prenatal and neonatal factors, Allergic diseases, School children

## Abstract

**Background:**

Allergic diseases, such as asthma, dermatitis, rhinitis, and eczema, are highly prevalent in Chinese school children. Environmental factors, including air pollution and automobile exhaust, play an important role in the etiology of these diseases. However, prenatal and neonatal factors, such as gender, maternal diseases during pregnancy, and premature birth, may also be associated with allergic disease occurrence. The objective of this study was to explore prenatal and neonatal factors that are involved in the development of allergic diseases among primary and middle school students in Guangzhou, China.

**Methods:**

A cross-sectional survey was launched by the Health Promotion Centre for Primary and Secondary Schools of the Guangzhou Municipality in October 2017. All primary and middle school students in Guangzhou were notified to participate in the questionnaire online under the direction of their parents. The results of the physical examination were reported by the schools’ medical department. The results of the questionnaire were collected and analyzed by the researchers. The prevalence of asthma, allergic rhinitis, allergic dermatitis, and eczema was identified.

**Results:**

Based on reported 183,449 questionnaires and medical records, the data indicate that the sex, birth weight, neonatal feeding type, delivery mode, and students’ father smoking status were significantly associated with the prevalence of all four allergic diseases in primary and middle school children. In further stratified analyses of the children with normal birth weight (2500–4000 g) and without any maternal diseases during pregnancy, the factors of male sex, high birth weight, cesarean delivery, and father smoking status all increased the risk of asthma, dermatitis, rhinitis, and eczema. Also, unlike exclusive breastfeeding, breast plus formula feeding increased these risks, but pure formula feeding had the opposite effect.

**Conclusion:**

Prenatal and neonatal factors, including male sex, high birth weight, cesarean delivery, only child, and father smoking status are associated with the risks of allergic diseases in school children.

## Background

Allergic diseases, such as asthma, dermatitis, rhinitis, and eczema, have become a severe clinical and public health problem, both worldwide and in the Guangzhou area. Asthma is a chronic inflammation of the airways that is often accompanied by extensive and variable airflow obstruction [1]. Allergic dermatitis is an inflammatory reaction that occurs at a contact site or part of the skin or mucous membrane after a single or multiple exposures to an exogenous substance [2]. Allergic rhinitis is characterized by at least two nasal symptoms: rhinorrhoea, blockage, sneezing or itching [3]. Eczema is a chronic, relapsing illness characterized by skin lesions [4]. These four allergic diseases are highly prevalent in Chinese school children, and the prevalence rates of asthma, allergic dermatitis, allergic rhinitis, and eczema are about 10.8, 0.5, 10.8, and 4.8%–15.8%, respectively [5–8].

Environmental factors, such as air pollution and automobile exhaust, play an important role in the etiology of these four allergic diseases. Tobacco smoke, due to parental smoking, is considered a major indoor pollutant for children with allergic diseases [9–12]. In addition, prenatal and neonatal factors, such as gender, maternal diseases during pregnancy, and premature birth, may be associated with allergic disease occurrence. It is known that the prevalence of asthma in adult women has increased compared with men [13, 14], and the prevalence of allergic dermatitis is significantly higher in girls than in boys [15]. Compared with full-term born children, premature children have a higher risk of allergic diseases, such as severe asthma and atopic dermatitis [16–18]. Meta-analysis has shown that low birth weight significantly increased the risk of asthma in children [19]. Breastfeeding is considered a protective measure against the development of allergic diseases, at least for neonates [20–23]. In addition, cesarean section (CS) and instrumental delivery might render the baby more susceptible to allergic diseases, most likely because it disrupts the mother-to-newborn transmission of microbes [24–28]. Maternal diseases during pregnancy are known to have a negative effect on fetal development. Moreover, maternal hypertension and thyroid dysfunction during pregnancy have been associated with a high prevalence of allergic diseases in the offspring [29–31]. Thus, prenatal and neonatal factors have a significant impact on the development of childhood diseases; however, further research is warranted in order to fully understand how these factors influence allergic diseases in children [32].

The purpose of our study was to systematically address the effects of various prenatal and neonatal factors on the development of allergic diseases in primary and secondary school students in Guangzhou, China. Based on a large population survey in Guangzhou primary and middle school, our study may provide insights into, and evidence of, the association between prenatal and neonatal factors and childhood allergic diseases in Southern area of China.

## Methods

### Data source

This study was approved by the ethics committee of The Third Affiliated Hospital of Guangzhou Medical University [2017(No.128)], and studies involving human subjects were conducted in accordance with the Declaration of Helsinki guidelines.

A cross-sectional survey design was applied, and all primary and middle schools in Guangzhou, China were invited to participate. The health survey was launched and supervised by the Health Promotion Centre for Primary and Secondary Schools of the Guangzhou Municipality, which is responsible for monitoring the health status of students from primary and secondary schools in Guangzhou. All primary and secondary school students in Guangzhou were notified by their school to participate in the survey in October 2017.

This health survey required participated children and parents jointly filled out the online questionnaire according to their own situation and directly submitted the questionnaire (see the Additional file 1). The questionnaire collected comprehensive data regarding the birth, and relevant prenatal, neonatal, and family information regarding the child, including birth weight, sex, neonatal feeding, delivery, delivery date, maternal diseases during pregnancy, parents’ education, parental smoking, and average household monthly income per person. At the time of the questionnaire or ever diagnosed diseases including asthma, dermatitis, rhinitis, and eczema were self-reported by the participants according to their out-patient clinical records.

According to the 2017 Guangzhou Education Statistics Handbook, the number of primary and secondary school students in 2017 was 1,514,122. The survey covered 11 administrative districts, including 991 schools. The first page of the questionnaire stated that the results of the health questionnaire would be used for health research.

### Statistical methods

The characteristics of the participants were reported as mean (SD, standard deviation) for continuous variables and frequency (proportion) for categorical variables. The prevalence (95% confidence interval (CI)) of allergic diseases was estimated by the categories of the participants’ characteristics. The prevalence of categories was compared using logistic regression. Multiple logistic regression analysis was performed in order to detect potential risk factors of allergic diseases. The participants who were singletons with normal birth weights (2500–4000 g) and whose mother had no diseases during pregnancy were included in the regression analysis. Questionnaires with missing data were not used for analysis. Variables with *P* < 0.05 in simple regression analysis were included in the multiple regression model. All *P* values were based on two-sided tests (*P* < 0.05 was considered significant). Statistical analyses were performed using SAS version 9.4 (SAS Institute Inc., Cary, NC, USA).

## Results

The health survey was launched and supervised by the Health Promotion Centre for Primary and Secondary Schools of the Guangzhou Municipality. All primary and secondary school students in Guangzhou City (1479 schools and 1,514,122 students) were invited to participate in this study. There were 991 schools, and a total of 253,301 students participated in the survey. Participants were from 11 districts within Guangzhou city, including Liwan, Yuexiu, Haizhu, Tianhe, Baiyun, Huangpu, Panyu, Huadu, Nansha, Chonghua, and Zengcheng. Their ages ranged from 6 to 18 years old. The final collected questionnaire number was 253,301, and the number of questionnaires with missing data was 69,852. The response rate was 16.7%.

Among the students participating in the study, 3449 (1.37%), 13,819 (5.50%), 10,260 (4.08%), and 11,484 (4.63%) experienced asthma, allergic dermatitis, allergic rhinitis, and eczema, respectively. The mean (standard deviation, SD) of birth weight students was 2990 (420) g. Males accounted for 53.7%. The percentage of singletons participating was 38.6%. The three types of neonatal feeding — breastfeeding, breast and formula feeding, and formula feeding — accounted for 44.0, 38.9, and 17.1%, respectively. Vaginal delivery accounted for 58.8% and CS accounted 41.2% of the completed surveys. According to the results, 27.6% of the children were full-term born, 24.3% were post-term born, and 48.2% were preterm born. Only 12.6% reported major maternal diseases during pregnancy. The prevalence of smoking by the father was 50.7%, with 7.95% of whom were quit > 1 year before the survey, 2.34% of whom quit < 1 year before the survey, and 40.50% as current smokers. The prevalence of smoking among the mothers was 0.9%, with 99.1% reporting as never having smoked. (Table 1).

**Table 1:** Characteristics of participants

Characteristics	All	Non-case group*	Case group*	*P*-value§
Total, *n* (%)	183,449 (100)	158,305 (86.3)	25,144 (13.7)	/
Grade, *n* (%)				< 0.001†
1–6	136,771 (74.6)	117,450 (74.2)	19,321 (76.8)	
7–9	33,013 (18.0)	29,014 (18.3)	3999 (15.9)	
10–12	13,665 (7.45)	11,841 (7.48)	1824 (7.25)	
Age, Years, *n* (%)^#^				< 0.001†
6–10	114,026 (62.2)	97,603 (61.7)	16,423 (65.3)	
11–15	59,374 (32.4)	51,961 (32.8)	7413 (29.5)	
> 15	10,049 (5.48)	8741 (5.52)	1308 (5.20)	
Mean (SD)	9.94 (2.99)	9.97 (3.00)	9.76 (2.96)	< 0.001¶
Sex, *n* (%)				
Male	98,565 (53.7)	83,995 (53.1)	14,570 (58.0)	< 0.001‡
Female	84,884 (46.3)	74,310 (46.9)	10,574 (42.1)	
Birth weight, kg, Mean (SD)	2.99 (0.42)	2.98 (0.42)	3.02 (0.43)	< 0.001¶
Neonatal feeding, *n* (%)				< 0.001‡
Breast feeding	80,718 (44.0)	70,723 (44.7)	9995 (39.8)	
Breast+ formula feeding	71,363 (38.9)	60,184 (38.0)	11,179 (44.5)	
Formula feeding	31,368 (17.1)	27,398 (17.3)	3970 (15.8)	
Delivery, *n* (%)				< 0.001‡
Vaginal delivery	107,830 (58.8)	94,232 (59.5)	13,598 (54.1)	
Cesarean	75,619 (41.2)	64,073 (40.5)	11,546 (45.9)	
Delivery date, *n* (%)				< 0.001‡
On the due date	50,595 (27.6)	45,627 (28.8)	4968 (19.8)	
Overdue	44,520 (24.3)	37,855 (23.9)	6665 (26.5)	
Before the due date	88,334 (48.2)	74,823 (47.3)	13,511 (53.7)	
Diseases in pregnancy, *n* (%)				
Hypertension	3005 (1.64)	2472 (1.56)	533 (2.12)	< 0.001‡
Diabetes	4290 (2.34)	3329 (2.10)	961 (3.82)	< 0.001‡
Intrahepatic cholestasis	486 (0.26)	383 (0.24)	103 (0.41)	< 0.001‡
Hypothyroidism	590 (0.32)	470 (0.30)	120 (0.48)	< 0.001‡
Hyperthyroidism	760 (0.41)	626 (0.40)	134 (0.53)	0.002‡
Anemia	13,515 (7.37)	10,701 (6.76)	2814 (11.2)	< 0.001‡
Viral hepatitis	1950 (1.06)	1604 (1.01)	346 (1.38)	< 0.001‡
Other	1331 (0.73)	968 (0.61)	363 (1.44)	< 0.001‡
Any disease above	23,175 (12.6)	18,434 (11.6)	4741 (18.9)	< 0.001‡
Only child, *n* (%)	70,832 (38.6)	59,201 (37.4)	11,631 (46.3)	< 0.001‡
One or both parents’ education> 12 years, *n* (%)	133,304 (72.7)	113,317 (71.6)	19,987 (79.5)	< 0.001‡
Father smoking, *n* (%)				0.129†
Never smoked	90,370 (49.3)	78,172 (49.4)	12,198 (48.5)	
Quit for > 1 year	14,584 (7.95)	12,410 (7.84)	2174 (8.65)	
Quit for < 1 year	4291 (2.34)	3698 (2.34)	593 (2.36)	
Current smoking	74,204 (40.5)	64,025 (40.4)	10,179 (40.5)	
Mother smoking, n (%)				0.519†
Never smoked	181,814 (99.1)	156,903 (99.1)	24,911 (99.1)	
Quit for > 1 year	672 (0.37)	575 (0.36)	97 (0.39)	
Quit for < 1 year	218 (0.12)	189 (0.12)	29 (0.12)	
Current smoking	745 (0.41)	638 (0.40)	107 (0.43)	
Average Household monthly income per person, US$, *n* (%)				< 0.001†
< 735	61,248 (33.4)	54,362 (34.3)	6886 (27.4)	
735–1764	74,842 (40.8)	64,471 (40.7)	10,371 (41.3)	
> =1764	47,359 (25.8)	39,472 (24.9)	7887 (31.4)	

*SD*: Standard deviation; 1USAdollar = 6.80RMB

^#^: The mean age of school grade 1 is six years old

* Case group included children who had allergic asthma, allergic dermatitis, allergic rhinitis and eczema, and children without above cases were included in the non-case group

§ P value was for comparing the differences between case group and non-case group

† Ordinal logistic regression was used for the comparison

‡ Chi-squared test was used for the comparison

¶ Two sample *t* test was used for the comparison

The prevalence (95% confidence interval, CI) of asthma, allergic dermatitis, allergic rhinitis, and eczema was higher (*P* < 0.001) in boys than girls: 1.94% (1.85, 2.03%) vs. 1.09% (1.02, 1.16%), 6.80% (6.64, 6.96%) vs. 5.95% (5.79, 6.11%), 4.50% (4.37, 4.63%) vs. 3.74% (3.61, 3.87%), and 5.51% (5.36, 5.65%) vs. 4.71% (4.57, 4.85%), respectively (Table 2). The prevalence (95% CI) of asthma in neonatal babies being breastfed only was lower [1.31% (1.24, 1.38%)] than that in those being breastfed and formula fed together (mixed feeding) [1.79% (1.69, 1.88%), *P* < 0.001], and lower than that in those being formula fed only [1.61% (1.47, 1.75%), *P* < 0.001]. The same direction of differences in the prevalence (95% CI) of allergic dermatitis, allergic rhinitis, and eczema was found comparing breastfeeding only [5.61% (5.46, 5.77%), 3.61% (3.48, 3.74%), 4.83% (4.68, 4.97%), respectively] with mixed feeding [7.69% (7.49, 7.88%), 4.78% (4.63, 4.94%), 5.71% (5.54, 5.88%), respectively, all *P* < 0.001] and formula feeding only [5.52% (5.27, 5.78%), 4.11% (3.89, 4.33%), 4.65% (4.42, 4.88%), respectively, all *P* < 0.001]. The prevalence (95% CI) of asthma [1.88% (1.78, 1.98%)], allergic dermatitis [7.27% (7.08, 7.45%)], allergic rhinitis [4.77% (4.62, 4.92%)], and eczema [5.47% (5.31, 5.63%)] was higher (*P* < 0.001) in students born by CS than that in students born by vaginal delivery [1.31% (1.24, 1.38%), 5.80% (5.66, 5.94%), 3.72% (3.60, 3.83%), 4.91% (4.78, 5.04%), respectively, all *P* < 0.001]. Students whose mother had maternal pregnancy diseases had higher (all *P* < 0.001) prevalence of allergic diseases than students whose mother had no maternal pregnancy disease (Table 2). Students who were singletons had higher prevalence (95% CI) of asthma [1.95% (1.84, 2.05%)], allergic dermatitis [7.90% (7.70, 8.10%)], allergic rhinitis [5.08% (4.91, 5.24%)], and eczema [6.00% (5.83, 6.18%)] than students who had siblings [1.29% (1.23, 1.36%), 5.47% (5.33, 5.60%), 3.57% (3.46, 3.68%), 4.59% (4.47, 4.72%), respectively, all *P* < 0.001].

**Table 2:** Prevalence (95% CI) of allergic diseases by categories of the participants’ characteristics (*N* = 183,449) *^#^

	Allergic asthma§	Allergic dermatitis†	Allergic rhinitis‡	Eczema¶
All	1.55 (1.49, 1.60)	6.41 (6.29, 6.52)	4.15 (4.06, 4.24)	5.14 (5.04, 5.24)
Grade				
1–6	1.47 (1.41, 1.53)	6.94 (6.80, 7.07)	4.19 (4.08, 4.30)	5.21 (5.09, 5.33)
7–9	1.70 (1.56, 1.83)**	4.68 (4.45, 4.91)***	4.02 (3.81, 4.23)	4.71 (4.48, 4.94)***
10–12	1.96 (1.72, 2.19)***	5.27 (4.90,5.65)***	4.08 (3.75, 4.41)	5.43 (5.05, 5.81)
Age, Years				
6–10	1.45 (1.38, 1.52)	7.25 (7.10, 7.40)	4.20 (4.09, 4.32)	5.28 (5.15, 5.41)
11–15	1.66 (1.56, 1.76)***	5.06 (4.88, 5.23)***	4.06 (3.91, 4.22)	4.83 (4.66, 5.01)***
> 15	1.99 (1.72, 2.26)***	4.81 (4.39, 5.23)***	4.09 (3.70, 4.48)	5.32 (4.88, 5.76)
Sex				
Female	1.09 (1.02, 1.16)	5.95 (5.79, 6.11)	3.74 (3.61, 3.87)	4.71 (4.57, 4.85)
Male	1.94 (1.85, 2.03)***	6.80 (6.64, 6.96)***	4.50 (4.37, 4.63)***	5.51 (5.36, 5.65)***
Neonatal feeding				
Breast feeding	1.31 (1.23, 1.39)	5.61 (5.46, 5.77)	3.61 (3.48, 3.74)	4.83 (4.68, 4.97)
Breast + formula feeding	1.79 (1.69, 1.88)***	7.69 (7.49, 7.88)***	4.78 (4.63, 4.94)***	5.71 (5.54, 5.88)***
Formula feeding	1.61 (1.47, 1.75)***	5.52 (5.27, 5.78)	4.11 (3.89, 4.33)***	4.65 (4.42, 4.88)
Delivery				
Vaginal delivery	1.31 (1.24, 1.38)	5.80 (5.66, 5.94)	3.72 (3.60, 3.83)	4.91 (4.78, 5.04)
Cesarean	1.88 (1.78, 1.98)***	7.27 (7.08, 7.45)***	4.77 (4.62, 4.92)***	5.47 (5.31, 5.63)***
Diseases in pregnancy				
Hypertension				
No	1.52 (1.46, 1.58)	6.36 (6.25, 6.47)	4.13 (4.04, 4.22)	5.11 (5.01, 5.21)
Yes	3.17 (2.54, 3.79)***	9.10 (8.07, 10.1)***	5.59 (4.77, 6.42)***	6.87 (5.97, 7.78)***
Diabetes				
No	1.50 (1.44, 1.56)	6.26 (6.15, 6.37)	4.08 (3.99, 4.17)	5.09 (4.99, 5.19)
Yes	3.48 (2.93, 4.02)***	12.5 (11.5, 13.5)***	7.12 (6.35, 7.90)***	7.25 (6.47, 8.02)***
Intrahepatic cholestasis				
No	1.54 (1.48, 1.59)	6.39 (6.28, 6.50)	4.14 (4.05, 4.23)	5.13 (5.03, 5.23)
Yes	4.54 (2.68, 6.39)***	11.3 (8.52, 14.2)***	9.05 (6.50, 11.6)***	9.11 (6.54, 11.7)***
Hypothyroidism				
No	1.54 (1.48, 1.59)	6.39 (6.28, 6.50)	4.14 (4.05, 4.23)	5.13 (5.03, 5.23)
Yes	4.76 (3.04, 6.48)***	10.7 (8.23, 13.2)***	8.32 (6.09, 10.6)***	8.72 (6.43, 11.0)***
Hyperthyroidism				
No	1.53 (1.48, 1.59)	6.40 (6.29, 6.51)	4.14 (4.05, 4.23)	5.13 (5.03, 5.23)
Yes	4.61 (3.12, 6.10)***	8.30 (6.34, 10.3)*	7.51 (5.63, 9.38)***	6.94 (5.12, 8.76)*
Anemia				
No	1.50 (1.45, 1.56)	6.06 (5.95, 6.18)	3.94 (3.84, 4.03)	4.95 (4.85, 5.06)
Yes	2.08 (1.84, 2.32)***	10.7 (10.2, 11.2)***	6.87 (6.45, 7.30)***	7.49 (7.04, 7.93)***
Viral hepatitis				
No	1.53 (1.48, 1.59)	6.38 (6.27, 6.49)	4.13 (4.04, 4.22)	5.11 (5.01, 5.22)
Yes	2.77 (2.04, 3.50)***	8.83 (7.57, 10.1)***	6.11 (5.05, 7.17)***	7.33 (6.17, 8.50)***
Other				
No	1.53 (1.47, 1.58)	6.35 (6.24, 6.46)	4.10 (4.01, 4.19)	5.11 (5.01, 5.21)
Yes	4.22 (3.14, 5.30)***	13.8 (12.0, 15.7)***	11.3 (9.59, 13.0)***	9.12 (7.56, 10.7)***
Any disease above				
No	1.42 (1.36, 1.48)	5.82 (5.71, 5.94)	3.79 (3.70, 3.88)	4.83 (4.73, 4.94)
Yes	2.43 (2.23, 2.63)***	10.4 (10.0, 10.8)***	6.65 (6.33, 6.97)***	7.26 (6.93, 7.60)***
Only child				
No	1.29 (1.23, 1.36)	5.47 (5.33, 5.60)	3.57 (3.46, 3.68)	4.59 (4.47, 4.72)
Yes	1.95 (1.84, 2.05)***	7.90 (7.70, 8.10)***	5.08 (4.91, 5.24)***	6.00 (5.83, 6.18)***
Father smoking				
Never smoked	1.56 (1.47, 1.64)	6.45 (6.29, 6.61)	3.99 (3.87, 4.12)	5.00 (4.86, 5.14)
Quit for > 1 year	1.86 (1.64, 2.08)**	6.61 (6.21, 7.02)	4.58 (4.24, 4.92)***	5.66 (5.28, 6.04)***
Quit for < 1 year	1.63 (1.25, 2.01)	6.11 (5.40, 6.83)	4.01 (3.43, 4.60)	5.43 (4.75, 6.11)
Current smoking	1.47 (1.38, 1.56)	6.33 (6.15, 6.50)	4.27 (4.12, 4.41)**	5.19 (5.03, 5.35)

*CI* Confidence Interval

*: prevalence (95% CI) (* *P* < 0.05, ** *P* < 0.01 and *** *P* < 0.001 indicating the significance of comparing each of other categories with the first category as the reference group

^#^: Two proportion Z test was used for comparing prevalence of allergic disease in categories of the participants’ characteristics with the first category as the reference group

§: 2,832/183,162 (1.55%), 287 missing values;

†: 11,731/183,142 (6.41%), 307 missing values;

‡: 7,607/183,238 (4.15%), 211 missing values;

¶: 9,352/181,997 (5.14%), 1452 missing values

Table 3 summarizes the results of four multiple logistic regression models for asthma, allergic dermatitis, allergic rhinitis, and eczema (*N* = 149,726). The participants in the model were singletons with normal birth weight (2500–4000 g) and whose mother had no diseases during pregnancy, as both birth weight and maternal pregnancy diseases are known factors that influence fetal growth and long-term diseases. With the age of students, the risk of allergic asthma [OR (95% CI): 1.04 (1.03, 1.06), *P* < 0.001] increased, while the risk of allergic dermatitis [OR (95% CI: 0.93 (0.93, 0.94, *P* < 0.001)] and eczema [OR (95% CI: 0.99 (0.98, 0.99), *P* < 0.001] decreased. A higher risk of asthma, allergic dermatitis, allergic rhinitis, and eczema was detected in males compared with females [OR (95% CI): 1.78 ((1.62, 1.95), 1.09 (1.04, 1.14), 1.18 (1.12, 1.24), and 1.14 (1.09, 1.20), respectively, all *P* < 0.001], in students with greater weight [OR (95% CI): 1.29 (1.15, 1.44), 1.28 (1.21, 1.36), 1.22 (1.14, 1.31), and 1.09 (1.02, 1.16), respectively, all *P* < 0.001], and also in the students born by CS compared to students born by vaginal delivery [OR (95% CI): 1.32 (1.21, 1.44), 1.17 (1.12, 1.22), 1.25 (1.18, 1.32), and 1.07 (1.01, 1.12) respectively, all *P* < 0.001]. Compared to breastfeeding only, the risk of asthma, allergic dermatitis, allergic rhinitis, and eczema was higher for mixed feeding [OR (95% CI): 1.27 (1.16. 1.40), 1.32 (1.26, 1.38), 1.35 (1.27, 1.43), and 1.16 (1.11, 1.23), respectively, all *P* < 0.001]. We found that the smoking status of the students’ father was associated with the prevalence of allergic diseases as well, as students whose father quitted for > 1 year had a higher risk of asthma, allergic rhinitis, and eczema [OR (95% CI): 1.23 (1.06, 1.43), 1.12 (1.02, 1.24), and 1.19 (1.09, 1.30), respectively, all *P* < 0.001].

**Table 3:** Multiple Logistic regression model of potential risk factors of allergic diseases among participants (OR (95% CI) and *P* value were presented) (*N* = 149,726) *

Variable^#^	Allergic asthma	Allergic dermatitis	Allergic rhinitis	Eczema
Age, Year	1.04 (1.03, 1.06) < 0.001	0.93 (0.925, 0.94) < 0.001	/	0.99 (0.98, 0.995) 0.002
Male	1.78 (1.62, 1.95) < 0.001	1.09 (1.04, 1.14) < 0.001	1.18 (1.12, 1.24) < 0.001	1.14 (1.09, 1.20) < 0.001
Birth weight, kg	1.29 (1.15, 1.44) < 0.001	1.28 (1.21, 1.36) < 0.001	1.22 (1.14, 1.31) < 0.001	1.09 (1.02, 1.16) 0.008
Neonatal feeding				
Breast feeding	Reference	Reference	Reference	Reference
Breast+ formula feeding	1.27 (1.16, 1.40) < 0.001	1.32 (1.26, 1.38) < 0.001	1.35 (1.27, 1.43) < 0.001	1.16 (1.11, 1.23) < 0.001
Formula feeding	1.08 (0.95, 1.23) 0.230	0.89 (0.83, 0.95) < 0.001	1.11 (1.02, 1.20) 0.014	0.91 (0.84, 0.97) 0.007
Delivery				
Vaginal delivery	Reference	Reference	Reference	Reference
Cesarean	1.32 (1.21, 1.44) < 0.001	1.17 (1.12, 1.22) < 0.001	1.25 (1.18, 1.32) < 0.001	1.07 (1.01, 1.12) 0.012
Only child	1.32 (1.21, 1.44) 0.002	1.45 (1.38, 1.51) < 0.001	/	1.30 (1.24, 1.37) < 0.001
Father smoking				
Never smoked	Reference	/	Reference	Reference
Quit for > 1 year	1.23 (1.06, 1.43) 0.006	/	1.12 (1.02, 1.24) 0.020	1.19 (1.09, 1.30) < 0.001
Quit for < 1 year	1.10 (0.84, 1.45) 0.486	/	0.94 (0.78, 1.13) 0.520	1.11 (0.95, 1.29) 0.189
Current smoking	0.91 (0.83, 1.00) 0.038	/	1.06 (1.00, 1.12) 0.039	1.07 (1.02, 1.13) 0.007

*OR* Odds Ratio, *C*I Confidence Interval

*: The multiple Logistic regression was conducted among participants who were singletons with normal birth weight (2.5-4 kg) and whose mother had no pregnancy disorder during pregnancy

#: All variables in the Table 1 were included in the simple regression. Variables with *P* < 0.05 in the simple regression analysis were included in the multiple regression model. The results of simple regression analysis were not listed in the table

## Discussion

According to Developmental Origins of Health and Disease (DOHaD) theory, the development of childhood diseases may be affected by factors in prenatal and neonatal life [32, 33]. Several epidemiological studies have revealed that prenatal and neonatal factors, such as the delivery mode, feeding type, and pregnancy diseases, may alter the risks of childhood allergic diseases, including asthma, dermatitis, rhinitis, and eczema [9, 16, 34–38]. However, most of these studies have been conducted in Western countries, and further epidemiological studies covering different areas and populations are warranted. In the Guangzhou area of southern China, allergic diseases are prevalent in school children. Although environmental factors, such as indoor and outdoor smoke, have been widely discussed, prenatal and neonatal factors are largely omitted. Therefore, a retrospective survey in all Guangzhou primary and middle school students was launched to study the association between prenatal and neonatal factors and allergic disease prevalence in school students 6–17 years old.

Based on 183,449 reported questionnaires and medical records, this cross-sectional survey study revealed that a parent-reported history of asthma, dermatitis, rhinitis, and eczema was present in 1.55% (1.49, 1.60%), 6.41% (6.29, 6.52%), 4.15% (4.06, 4.24%), and 5.14% (5.04, 5.24%) of school children, respectively (Table 2). In this study, the prevalence of asthma and rhinitis was lower, while the prevalence of dermatitis and eczema was higher than the reported prevalence in China [5–8]. These differences may be due to the lower air pollution in the Guangzhou area relative to northern China, and to the wet and hot climate of this area, which might cause more risks of skin diseases.

In our study, epidemiological data supported that the sex, birth weight, neonatal feeding type, delivery mode, and the smoking status of the father were significantly associated with the prevalence of all four allergic diseases. In further stratified analyses of children with normal birth weight without any maternal pregnancy diseases, prenatal and neonatal factors, including male sex, high birth weight, cesarean delivery, only child, and smoking status of the father, all increased the risks of asthma, dermatitis, rhinitis, and eczema in primary and middle school children. Unlike breastfeeding alone, breast plus formula feeding increased the risk of allergic diseases, but pure formula feeding decreased the risks.

Previous studies have reported that the prevalence of four allergic diseases — asthma, dermatitis, rhinitis, and eczema — has gender disparity within different populations [13]. For instance, asthma prevalence and mortality are higher among adult women than men overall; however, the prevalence is higher among boys than girls 0–14 years of age. The reasons for the gender difference are not thoroughly understood, but have been linked to gender-specific responses to environmental or occupational exposures involving immunological and hormonal factors [13, 14]. Our study also supported that boys have a higher risk of asthma, dermatitis, rhinitis, and eczema in all age groups relative to girls. In the Aberdeen Schools Asthma Survey (ASAS) in Scotland, one of the longest-running pediatric epidemiology studies in the world, males were reported to have a higher risk of asthma [9]. A national register study in Finland assessed 1,018,302 children between 1991 and 2008 and found that the male sex predicted asthma medication in preterm children [16]. In Norwegian registries with prospectively collected data, the male gender showed an increased asthma risk [17]. In addition, aging increases the incidence of allergic diseases from our results. In the Scotland ASAS study, the odds of a parent-reported lifetime of asthma increased with age [9], which is consistent with our results.

Smoking is a common environmental factor that is a health hazard. Almost all epidemiologic studies reported, thus far, support the negative impact of environmental tobacco smoke or parental cigarette smoking on pediatric allergic diseases, such as asthma. In the Scotland ASAS study, parental smoking increased the risk of asthma and wheezing, but not eczema and hay fever [9]. In Finland, parental smoking was highly associated with childhood asthma [39], and maternal smoking predicted asthma medication in preterm children [16]. In a Japanese study of 36,888 surveyed, maternal indoor smoking significantly increased the risk of asthma development in children, and paternal smoking slightly increased the prevalence of asthma development in children, but was not statistically significant [12]. In the city of Guangzhou in China, the situation differs from other areas. First, the male smoking rate is reported to be as high as 48.4%, and most began smoking as teenagers. Although many fathers quit smoking after childbirth, the potential effects of smoking still exist; therefore, previous smoking status should be considered. Second, female smoking is rare, to such an extent that in this study 99.2% of the mothers never smoked; therefore, unlike other countries, maternal smoking in China may not be a significant factor for consideration. Thus, the impact of indoor smoke hazards may decrease, while outdoor environmental hazard exposure may increase. Our study supports that the smoking status of the father, particularly smoking vs. non-smoking, influences allergic diseases in children, which may reflect the epigenetic alterations during early lifetime.

Preterm birth and low birth weight are considered risk factors for major allergic diseases. In Japan, a recent cross-sectional study found no significant associations between low birth weight, preterm birth, and prevalence of wheezing, asthma, or eczema [11]. A meta-analysis of 13 cohort studies and 1,105,703 subjects found that the overall pooled risk ratio of asthma in low birth weight children was 1.162, and low birth weight significantly increased the risk of childhood asthma [19]. In Norwegian registries with prospectively collected data, preterm birth was associated with an increased risk of severe asthma and a decreased risk of severe atopic dermatitis [17]. Data from 700 families in the Netherlands supported that children with low birth weight tended to have a lower risk of any allergy, albeit not significant, and prematurity was a risk factor for asthma at six years of age [40]. Our study was retrospective, and parents only reported the situation of delivery as due date, overdue date, or before the due date, broad categories that can be correctly remembered by them. As preterm delivery is commonly believed to be a risk factor for allergic diseases, we only studied the children who had normal birth weight (2500–4000 g); hence, we excluded the preterm and overdue date children. Interestingly, in the normal birth weight children, the risk of allergies was adversely linked with birth weight, as higher birth weights had a higher risk for diseases.

Pregnancy diseases in mothers, including maternally related diseases, such as hyperemesis, hypertension, and preeclampsia, and uterus-related complications, such as antepartum hemorrhage, preterm contractions, and insufficient placenta, affect the growth of the fetus in the uterus and probably also the overall long-term health. In a population-based cohort study in Denmark involving 5,95,669 children, the incidence rate ratios of asthma were 1.1 and 1.12 for children born to mothers with hypothyroidism that was diagnosed before or after delivery. The highest risk was observed among children born to mothers with hypothyroidism diagnosed before delivery who did not receive thyroid hormone treatment during pregnancy [31]. A population-based four-year cohort study involving 2531 children born in Norway found only uterus-related, but not other pregnancy-related complications, increased the risk of bronchial obstruction, asthma, and allergic rhinitis [30]. In the Avon Longitudinal Study, hypertension before pregnancy was positively associated with wheezing and asthma [29]. Our data support that all pregnancy diseases, including preeclampsia and diabetes, increased the risk of allergic diseases in children.

The mode of delivery may affect outcomes, such as asthma and allergic disease, in children many years later. Recent meta-analyses have reported a 20% increase in the subsequent risk of asthma in children delivered by CS and a moderate risk increase for allergic rhinitis and asthma. Recent studies have reported that children born by CS have an increased risk of asthma, whereas others found no such association [10].. In a cross-sectional study of 672 children nested in a birth cohort evaluated at 6 years of age in Brazil, the parental history of asthma was an effect modifier in the association between CS, chronic rhinitis, and allergic rhinitis. CS increases the risk of chronic rhinitis and allergic rhinitis in children at six years of age that have a parental history of asthma [26]. Further investigations on the long-term effects of emergency and elective CS may include a systematic survey of all modes of delivery [12]. In a population-representative prospective birth cohort of 8327 Chinese children in Hong Kong, it was found that delivery by CS accounted for 27% of all births and was not clearly associated with hospitalizations for asthma or other wheezing disorders at 12 years of age compared to vaginal delivery. Similarly, there were no clear associations at 2 years of age or six years of age [41]. Our findings suggest an association between delivery by CS and childhood allergic disorders. However, if CS is stratified into an emergency CS and elected CS, the associations may vary and may not be biologically mediated.

Feeding type is another controversial factor for asthma. It is believed that breastfeeding is protective against allergic diseases, at least in infants. However, effects in older children, such as school students, are controversial. Exclusive breastfeeding for at least three months reduces the risk of atopic dermatitis, at least during infancy. No clear risk reduction was evident, however, for asthma, allergic rhinitis, positive allergen skin tests, or food allergies [20]. A systematic review suggested that a longer duration of breastfeeding (vs. a shorter duration) was associated with a reduced risk of asthma for children (5–18 years), particularly in medium- and low-income countries, and with a reduced risk of allergic rhinitis in children ≤5 years [21]. Our data show that breast plus formula feeding increased the risks of allergic diseases compared to exclusive breast milk, but pure formula feeding decreased the risks. One reason for this is that in the Guangzhou area, the formula is likely to be afforded by families with high incomes; therefore, formula feeding can be linked with socioeconomic status.

## Conclusion

Risk factors for allergic diseases have been explored for many years as the growing prevalence of allergic diseases in the world. This study collected the data from 183,449 surveys for investigation. The results indicate that, in school children without any maternal complications during pregnancy, prenatal and neonatal factors, including male sex, high birth weight, cesarean delivery, only child, and father’s smoking status all increased the risk of asthma, dermatitis, rhinitis, and eczema, but exclusive formula feeding decreased the risk. Therefore, this study reported the major prenatal and neonatal factors associated with allergic diseases in school children in the Southern area of China. Although the results overlap with other studies, it is still important that there is a very large population from a specific area participated in the study. Therefore, this study provides strong evidence to support known risk factors for allergic diseases in school children.

## Supplementary information

**Additional file 1 **Questionnaire**.** Description of data: an English language version of the questionnaire developed for this study

## Data Availability

Deidentified participant data are available upon reasonable requisition.

## Abbreviations

ASAS: Aberdeen Schools Asthma Survey
CI: Confidence Interval
CS: Caesarean Section
DOHaD: Developmental Origins of Health and Disease
HR: Hazard Ratio
IRR: Incidence Rate Ratios
OR: Odd Ratios
SD: Standard Deviation
SMS: Short Messaging Service
