# Supplementary material for: Prenatal and neonatal factors involved in the development of childhood allergic diseases in Guangzhou primary and middle school students
Source: BMC Pediatr. 2019 Dec 7;19:479. doi: 10.1186/s12887-019-1865-0 (PMC6898923; doi:10.1186/s12887-019-1865-0)
Supplement: Supplementary file 1 — Additional file 1 Questionnaire. Description of data: an English language version of the questionnaire developed for this study [file 12887_2019_1865_MOESM1_ESM.pdf]

Health monitoring questionnaire for primary and secondary school students

(This questionnaire is to be completed by parents/guardians and students)

Dear Parents/Students,

Hello! This survey aims to provide a basis for understanding students' lifestyle factors and health behaviors to help carry out health education in schools in the future. Please answer the following questions **truthfully** according to your own situation. There is no right or wrong answer, and your answers will be kept confidential. Please note that **the first and second sections should be completed by the parents.**

The multiple-choice questions in the questionnaire are **single-answer questions** unless otherwise indicated. Each question is very important; **please do not skip any question!**

School name: _______________ Grade: __________ Class:

Student's name: __________ Sex: [1] Male [2] Female Identification card number:

Birth date: ___ Year ____ Month ____ Day

Date of questionnaire completion: ___ Year ____ Month ____ Day

Section 1. Basic information (filled by a parent or guardian)

1. The personnel who completed the questionnaire:

[1] Father [2] Mother [3] Father and mother together [4] Grandfather/grandmother [5] Others

2. Birth weight of the student (your child): ___ *jin* (1 *jin* = ½ kilogram) ____ *liang* (1 *liang* = 50 grams) (Please also fill in the *liang* unit, e.g., 7 jin 5 liang)

Birth length: ___ cm

3. Mother's weight before pregnancy with this child: ___ kg, mother's height: ___ cm; mother's weight before birth of this child: ___ kg

4. What type of feeding (breast milk and/or formula) did the child have within six months of

birth?

[1] Exclusively breastfeeding [2] Mixed feeding (with breast milk and formula) [3] Bottle-feeding (with formula)

If the child was exclusively breastfed, he/she was fed until the month of age; if the child was mixed fed, he/she was breastfed until the month of age.

5. Method of your child's birth delivery: [1] Natural birth [2] Cesarean section

6. The child was born in: [1] A single birth [2] A twin birth [3] A higher-order birth (triplets or more)

7. How many days before or after the due date was your child born?

[1] Born on the expected due date

[2] Exceeded the expected due date by ____ week(s) ____ day(s)

[3] Born ____ week(s)____ day(s) earlier than the expected due date

8. Did the child's mother have any of the following medical conditions during pregnancy?

1) Gestational hypertension	[1] Yes [2] No		5) Gestational hyperthyroidism	[1] Yes [2] No
2) Gestational diabetes	[1] Yes [2] No		6) Gestational anemia	[1] Yes [2] No
3) Gestational cholestasis	[1] Yes [2] No		7) Viral hepatitis during pregnancy	[1] Yes [2] No
4) Gestational hypothyroidism	[1] Yes [2] No		8) Others	[1] Yes [2] No

9. Is the child an only child? [1] Yes [2] No

If not, how many siblings does the student have? ____ The child is the ____ child in the family.

10. The highest educational level of the child's father: ___ (Choose from the following options)

The highest educational level of the child's mother: ___ (Choose from the following options)

[1] Primary school or below [2] Middle school [3] High school/technical secondary school [4] Junior college/vocational college [5] Four-year college [6] Graduate school or above

11. Is the child’s father nearsighted? [1] Yes [2] No [3] Unknown

If your answer is “No” or “Unknown,” please skip to Question 12.

If your answer is “Yes,” the severity of the nearsightedness of the left eye is: [1] <−0.3 diopter (D) [2] −0.3 D to −0.6 D [3] >−0.6 D [4] Unknown

The severity of the nearsightedness of the right eye is: [1] <−0.3 D [2] −0.3 D to −0.6 D [3] >-0.6 D [4] Unknown

12. Is the child’s mother nearsighted? [1] Yes [2] No [3] Unknown

If your answer is “No” or “Unknown,” please skip to Question 13.

If your answer is “Yes,” the severity of the nearsightedness of the left eye is: [1] <−0.3 D [2] −0.3 D to −0.6 D [3] >−0.6 D [4] Unknown

The severity of the nearsightedness of the right eye is: [1] <−0.3 D [2] −0.3 D to −0.6 D [3] >−0.6 D [4] Unknown

13. Does the child’s father smoke? [1] Never smoked [2] Quit smoking more than a year ago [3] Quit smoking less than a year ago [4] Smokes

Does the child’s mother smoke? [1] Never smoked [2] Quit smoking more than a year ago [3] Quit smoking less than a year ago [4] Smokes

14. What is the per capita monthly household income in your family? (Note: Per capita monthly household income = Total monthly household income ÷ number of family members)

[1] 2,000 renminbi or below [2] 2,000 to <5,000 renminbi [3] 5,000 to <8,000 renminbi [4] 8,000 to <12,000 renminbi [5] 12,000 to <15,000 renminbi [6] ≥15,000 renminbi [7] Unknown

15. Has the child been diagnosed with any of the following diseases by a physician?

(1) **Hypertension**: [1] Yes [2] No; (2) **Dyslipidemia**: [1] Yes [2] No; (3) **Thalassemia**: [1] Yes [2] No;

(4) **Diabetes**: [1] Yes [2] No; If your answer is "Yes," which type of diabetes: [1] Type I [2] Type II [3] Other

(5) **Allergic diseases** (currently or ever diagnosed by doctors) :

Disease name	Yes/No	Age of first onset		Disease name	Yes/No	Age of first onset
**Asthma**	[1] Yes [2] No	years old		**Eczema**	[1] Yes [2] No	years old
**Allergic dermatitis**	[1] Yes [2] No	years old		**Urticaria**	[1] Yes [2] No	years old
**Allergic rhinitis**	[1] Yes [2] No	years old		**Other**	[1] Yes [2] No	years old

16. Have the child's parents, siblings, paternal grandparents, maternal grandparents, or uncle(s)/aunt(s) been diagnosed with any of the following diseases by physicians?

(1) Obesity: [1] Yes [2] No

(2) Hypertension: [1] Yes [2] No

(3) Diabetes: [1] Yes [2] No

(4) Heart disease(s) (e.g., coronary heart disease or myocardial infarction): [1] Yes [2] No

(5) Cerebrovascular disease(s) (e.g., cerebral thrombosis or stroke): [1] Yes [2] No

Section 2. Psychological behavior (completed by a parent or guardian)

17. Compared with children of the same age, do you feel that your child is hyperactive, impulsive, and inattentive?

[1] No [2] Mildly [3] Moderately [4] Strongly [5] Very severely

18. How do you rate your child's interpersonal skills compared with children at the same age?

[1] Very good [2] Quite good [3] Moderate [4] Quite bad [5] Very bad

19. Compared with children of the same age, your child's emotional control ability is:

[1] Very good [2] Quite good [3] Moderate [4] Quite bad [5] Very bad

20. Do you think that your child has the following emotional problems?

(1) Anxiety [1] No [2] Mild [3] Moderate [4] Strong [5] Very severe

(2) Depression [1] No [2] Mild [3] Moderate [4] Strong [5] Very severe

(3) Obsession [1] No [2] Mild [3] Moderate [4] Strong [5] Very severe

(4) Fearfulness [1] No [2] Mild [3] Moderate [4] Strong [5] Very severe

21. Does your child have any learning problems at school?

[1] No [2] A little [3] Moderate [4] Severe [5] Very severe

Section 3. Exercising and sleeping (completed by the student)

Questions 22 to 25 below only refer to activities that lasted for at least 10 minutes each time.

22. In the past seven days, you had ______ days of **high-intensity** exercise/physical activity (This type of exercise involves great effort, breathlessness, sweating, or tiring, such as running, basketball playing, soccer playing, swimming, moving heavy objects, rope jumping, roller skating, or taekwondo).

During these few days, these high-intensity exercises/physical activities were performed for an average of _____ hour(s) ______ minute(s) daily.

23. In the past seven days, you had ______ days of **moderate-intensity** exercise/physical activity (This type of exercise involves moderate effort, a bit of breathlessness and sweating, and a little tiring, such as cycling, table tennis, badminton, dancing, group exercise between classes, or moving light objects).

During these few days, these moderate-intensity exercises/physical activities were performed for an average of ______ hour(s) _____ minute(s) daily.

24. In the past seven days, you walked for ______ days (including walking at school and at home,

walking to and from school or for a trip, and walking for exercise).

During these days, you spent an average of ______ hour(s) _____ minute(s) daily walking.

25. In the past seven days, how much time did you spend on homework after school? An average of _____ hour(s) _____ minute(s) daily.

On watching television? An average of _____ hour(s) _____ minute(s) daily. On using a computer or playing video games? An average of _____ hour(s) _____ minute(s) daily.

On commuting to and from school (by public bus, subway, or car)? An average of _____ hour(s) _____ minute(s) daily.

26. How much time did you spend (on average) on daily outdoor activities in the past seven days?

[1] Less than one hour [2] One to less than two hours [3] Two to less than four hours [4] Four hours or more

27. How long (on average) have you been exposed to the sun daily in the past seven days?

[1] Less than 30 minutes [2] 30 to 59 minutes [3] One to two hours (not including two hours) [4] Two to four hours [5] More than four hours

28. You have smoked _____ days in the past 30 days, averaging _____ cigarettes per day. (If you do not smoke, please fill in “0”)

29. In the past week,

Question	**Monday to Friday**	**Saturday to Sunday**
1) At what time did you go to bed at night on average?	_______:________	_______:________
2) How long did you usually take to fall asleep every night?	________ minute(s)	________ minute(s)
3) At what time did you get up in the morning on average?	_______:________	________: ________
4) How long did you usually nap in the afternoon on average?	________ minute(s)	________ minute(s)

Section 4. Meals (filled in by student)

30. How many times did you eat fruit in the past seven days? _______ time(s)

Each time you had fruit, how many servings of fruit did you eat on average? _______ serving(s) (one serving = the size of an egg)

31. How many times did you eat vegetables in the past seven days (excluding pickles)? _______ time(s)

Each time you had vegetables, how many servings of vegetables did you eat on average? _______ serving(s) (one serving = an egg size)

32. On how many days did you eat coarse grains (e.g., corn, millet, sorghum, oats, or buckwheat) in the past seven days? _______ day(s)

On the day(s) when you had coarse grains, how many serving of coarse grains did you eat on average? _______ serving(s) (one serving = the size of an egg)

33. How many times did you eat fish or fish products in the past seven days? _______ time(s)

Each time you had fish or fish products, how many servings of fish or fish products did you eat on average? _______ serving(s) (one serving = the size of an egg)

34. How many times did you eat red meat or its products (e.g., pork, beef, and lamb) in the past seven days? _______ time(s)

Each time you had red meat or its products, how many servings of red meat or its products did you eat on average? serving(s) (one serving = the size of an egg)

35. Did you eat deep-fried foods (e.g., fried chicken, French fries, fried dough sticks, or Western-style fast foods from McDonald's, KFC, etc.) in the past seven days? _______ time(s)

36. How many sugary soft drinks (e.g., cola, Sprite, orange juice, Nutri-Express, or Red Bull) did you have in the past seven days? drink(s)

Each time you had sugary soft drinks, how many cup(s) did you consume on average?

_______ cup(s) (one cup is equivalent to 250 ml)

37. How many times did you have milk or dairy products (e.g., fresh milk, yogurt, or milk powder) in the past seven days? _______ time(s)

(1) Each time you had milk, what was the amount of milk you had? _______ ml (Single-serving milk box = 250 ml, single-serving yogurt bottle = 150 ml, one scoop of milk powder = 30 ml)

Please indicate your specific consumption of milk and dairy products in the past seven days (If you did not consume the product, please fill in "0")		
① Whole milk/formula _______ time(s), _______ ml each time<br>② Non-fat/low-fat milk/formula _______ time(s), _______ ml each time<br>③ Full-fat yogurt _______ time(s), _______ ml each time<br>③ Non-fat/low-fat yogurt _______ time(s), _______ ml each time		⑤ Milk-containing beverage (e.g., Yousuanru yogurt/ Nutri-Express) _______ time(s), _______ ml each time<br>⑥ Milk tea, milk shake _______ time(s), _______ ml each time<br>⑦ Cheese, milk curd _______ time(s), _____ _______ sheet each time (1 sheet = approximately 20 grams)<br>⑧ Ice cream, ice cream cone _______ time(s), _____ _______ serving(s) each time (one serving = approximately 80 grams)

38. Did you eat soybeans or their products (e.g., soybeans, soybean milk, tofu, soymilk, bean curd jelly, dried bean curd sticks, or fermented tofu)? ______ time(s)

(1) Each time you had liquid soy products (e.g., soybean milk, soymilk, or bean curd jelly), how many cup(s) did you consume? ______ cup(s) (one cup is equivalent to 250 ml)

(2) Each time you had solid soy products (e.g., soybeans, tofu, shredded tofu, dried bean curd sticks, or fermented tofu), how many serving(s) did you consume? ______ serving(s) (one serving = the size of an egg)

(3) Which types of the following beans and products did you eat often in the past seven days (you can choose more than one option)?

[1] Soybean [2] Mung bean [3] Adzuki bean (red bean) [4] Kidney bean [5] Broad bean [6] Pea [7] Tofu [8] Soybean milk [9] Soymilk [10] Bean curd jelly [11] Dried bean curd [12] Shredded tofu/bean curd skin [13] Dried bean curd sticks [14] Other(s) ______

39. How many cups of water did you drink daily in the past seven days on average?

______ cup(s) (one cup is equivalent to 250 ml)

40. On how many days did you eat breakfast in the past seven days? _______ day(s)

41. How would you compare your speed of finishing a meal with classmates of the same sex in the same class? [1] Slower than others [2] Same speed as others [3] Faster than others

On average, you need _______ minute(s) to finish one meal of lunch or dinner.

Your preference for food tends to be: [1] Salty (more salt) [2] Moderate (not salty and not bland) [3] Bland (less salt)

42. Are you a picky eater? [1] Yes [2] No

If your answer is “Yes,” at what age did you become a picky eater? _______ years old

Which of the following foods do you not like (can choose more than one option): [1] Milk and dairy products [2] Beans and their products [3] Grains and their products [4] Vegetables [5] Fruits [6] Meat [7] Dessert [8] Eggs [9] Other(s) __________
